# The DNA Damage Response and HIV-Associated Pulmonary Arterial Hypertension

**DOI:** 10.3390/ijms21093305

**Published:** 2020-05-07

**Authors:** Ari Simenauer, Eva Nozik-Grayck, Adela Cota-Gomez

**Affiliations:** 1 Department of Medicine Division of Pulmonary Sciences and Critical Care Medicine, University of Colorado Anschutz Medical Campus, Aurora, CO 80045, USA; ari.simenauer@cuanschutz.edu; 2 Cardiovascular Pulmonary Research Labs and Pediatric Critical Care Medicine, University of Colorado Denver, Pediatric Critical Care Medicine, Aurora, CO 80045, USA; eva.grayck@cuanschutz.edu

**Keywords:** HIV, pulmonary arterial hypertension, endothelial, DNA damage, Tat, Nef

## Abstract

The HIV-infected population is at a dramatically increased risk of developing pulmonary arterial hypertension (PAH), a devastating and fatal cardiopulmonary disease that is rare amongst the general population. It is increasingly apparent that PAH is a disease with complex and heterogeneous cellular and molecular pathologies, and options for therapeutic intervention are limited, resulting in poor clinical outcomes for affected patients. A number of soluble HIV factors have been implicated in driving the cellular pathologies associated with PAH through perturbations of various signaling and regulatory networks of uninfected bystander cells in the pulmonary vasculature. While these mechanisms are likely numerous and multifaceted, the overlapping features of PAH cellular pathologies and the effects of viral factors on related cell types provide clues as to the potential mechanisms driving HIV-PAH etiology and progression. In this review, we discuss the link between the DNA damage response (DDR) signaling network, chronic HIV infection, and potential contributions to the development of pulmonary arterial hypertension in chronically HIV-infected individuals.

## 1. Introduction

The continued development and global implementation of combinatorial anti-retroviral therapy, widely known as highly active anti-retroviral therapy (HAART), has been largely successful in reducing the burden of deadly opportunistic lung infections in immunocompromised HIV-infected patients. Concomitant with the increased longevity afforded by HAART, however, has been a dramatic increase in the prevalence of non-infectious cardiopulmonary disease, and pulmonary arterial hypertension (PAH) is among the most prevalent of these diseases associated with long term HIV infection [1]. PAH, defined as a mean pulmonary arterial pressure (MPAP) of >20 Hg, a mean pulmonary artery wedge pressure (PAWP) of ≤15 mm Hg, and a pulmonary vascular resistance (PVR) of ≥3 wood units, is characterized by the progressive obstruction of the small pulmonary arteries, resulting in increased pulmonary resistance and right ventricular load, ventricular hypertrophy, and frequently, death. Since the first prospective studies in the early 1990s, HIV has become a well-established and independent risk factor for the development of PAH, with an estimated prevalence of ~1:200, compared to ~1:1,000,000 in the general population. Strikingly, numerous modern multinational studies have established the prevalence of HIV-PAH to be equivalent to the rates observed in the pre-HAART era (0.5%), suggesting that the development of HIV-PAH is irrespective of the implementation of anti-retroviral therapy [2]. Due to relatively poor screening practices in underdeveloped countries, as well as the difficult nature of PAH diagnosis by means of right heart catheterization, the true prevalence of HIV-PAH has likely remained under-estimated, and improved diagnostic and screening practices continue to reveal HIV infection as one of the most common causes of PAH worldwide [3]. HIV-PAH, like idiopathic PAH (iPAH), involves the progressive remodeling of the small pulmonary arteries, characterized by neo-intimal endothelial hyperplasia, medial smooth muscle cell hypertrophy and arterial muscularization [4]. Despite these similarities, the exact mechanisms by which HIV drives the progression of PAH remain poorly defined. Understanding how interactions between HIV and the host at the cellular/molecular interface drive the progression of PAH represents a unique opportunity to shed light on a relatively enigmatic disease process and may indeed provide novel insights into not only HIV-PAH pathogenesis, but idiopathic PAH as well. Despite the established prevalence of HIV-PAH, the exact mechanisms by which HIV infection contributes to the development of cellular pathologies associated with PAH have remained elusive. Here, we discuss the role of DNA damage response (DDR) signaling, with an emphasis on DNA damage checkpoint induction, in the molecular pathophysiology of HIV-PAH.

## 2. HIV Factors in PAH

The fact that the prevalence of HIV-PAH in the age of HAART remains consistent is a surprising epidemiological observation. Additionally, none of the cell types most heavily involved in the pathogenesis of PAH are known to be productively infected by HIV, suggesting indirect mechanisms by which the virus may elicit pathological cellular phenotypes in pulmonary vascular arterial structures. Indeed, HIV transgenic animal models exhibit pulmonary arterial remodeling and increased vascular resistance, demonstrating that a replication competent virus is not necessary for development of a PAH phenotype [5,6]. These findings have led the field to focus on a number of soluble HIV factors that are known to be released into circulation during HIV infection, the most heavily implicated factors being negative factor (Nef), glycoprotein (Gp120) and the transactivator of transcription (Tat). HIV-PAH pathogenesis is a complex and somewhat opaque disease process, the etiology of which is likely multifactorial in terms of viral adaptation, host genetic susceptibility, and numerous environmental factors. In recent decades, genetic instability and DNA damage in PAH have become recognized as important hallmarks of PAH [7]. HIV employs an arsenal of viral factors, which elicit favorable conditions to viral replication, including the modulation of host DNA damage response (DDR) pathways [8]. The impairment of many of these pathways elicits anti-apoptotic and pro-proliferative cellular phenotypes, favoring viral replication and long-term persistence [9,10]. Here, we discuss HIV factor mediated dysregulation of the DDR in bystander cells involved in PAH pathogenesis, and the potential implications for their contribution to the development of HIV-PAH.

## 3. The DNA Damage Response

The DNA damage response is a multi-system signaling network which, at its core, is responsible for maintaining both immediate and abiding cellular genomic stability. DNA is subjected to constant chemical and physical stress, and the continuous maintenance of genomic integrity by cellular safeguards is required to prevent an accumulation of deleterious mutations. DNA is prone to alterations by numerous mechanisms, including single base alterations via spontaneous deamination/depurination of deoxyribonucleosides, as well as two base alterations and strand cross linkage [11]. Less innocuous DNA lesions include single strand chain breakage and, the most detrimental DNA lesion, double strand DNA breaks. DNA damage is often the result of endogenous cellular processes including metabolic production of reactive oxygen species, DNA replication, and transcriptional processes [12]. Exogenous DNA damaging sources including ultra-violet light, ionizing radiation, and foreign chemical electrophiles additionally contribute to all forms of DNA damage. The continuous barrage of genetically damaging factors, both endogenous and external, have necessitated the evolution of numerous mechanisms for DNA damage sensing and repair, which are critical to the maintenance and preservation of cellular genomic integrity. Defects in any component of this intricate system can result in genomic instability and the accumulation of chromosomal abnormalities.

**Figure 1:**
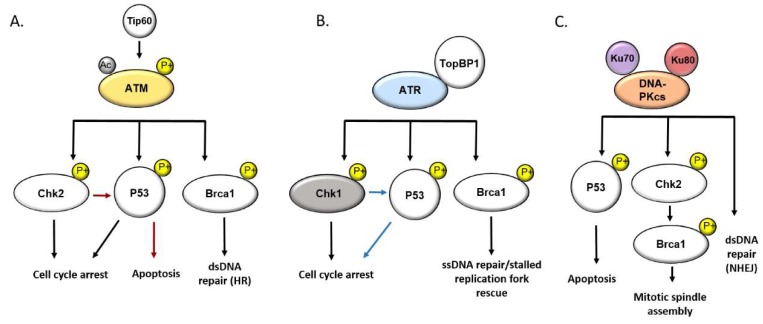
Simplified diagram of the dynamic and interconnected DNA damage response network orchestrated by the signal transducing kinases ATM, ATR, and DNA-PKcs: (**A**) Tip60 acetylates ATM at lysine residue 3016, resulting in auto phosphorylation and activation of ATM kinase monomers. ATM phosphorylation of Chk2 and P53 leads to induction of cell cycle arrest, while ATM phosphorylation of Brca1 initiates double stranded DNA repair through homologous recombination (HR). Chk2 phosphorylation of P53 independent of ATM leads to apoptosis. (**B**) ATR and TopBP1 localize to sites of DNA Damage and replicative stress, where ATR phosphorylates effectors substrates. ATR mediated activation of Chk1 and P53 induce cell cycle arrest, while phosphorylation of Brca1 augments DNA end resection and single stranded DNA break repair by HR. Phosphorylation of P53 by activated Chk1 further augments cell cycle arrest. (**C**) DNA-PK stabilizes P53 in response to excessive genotoxic stress, inducing apoptosis. The ku70 and Ku80 subunits of the DNA-PK complex act as scaffolding adaptors for DNA-PKcs and recruitment to dsDNA lesions, where DNA-PKcs facilitates DNA repair through the error prone non-homologous end joining pathway.

The DDR goes beyond the simple recognition and repair of DNA lesions. DDR signaling is a hierarchical and interconnected network that is critical to cell cycle regulation via checkpoint mediated arrest, the induction of cellular senescence, and programmed cell death. Under homeostatic conditions, the accumulation of irreparable DNA lesions results most often in cell death through induction of apoptosis, autophagy, or necrosis [13]. However, the notion of the DDR as a simple and direct response to DNA damage alone is an oversimplification of a diverse and interconnected system of signaling pathways that respond to various stressors in order to maintain cellular homeostasis. The kinases ataxia telangiectasia mutated (ATM), ataxia telangiectasia and RAD3 related (ATR), and DNA-protein kinase (DNA-PK) are master regulators of the DDR and serve as central signaling hubs between DNA damage sensing and effector substrate signaling, resulting in appropriate cell cycle arrest and/or ultimate cell survival, progression to senescence, or cell death [14]. Given the functionality of their target substrates, which includes CHK1, CHK2, BRCA1, and 53BP1, it is unsurprising that research in recent years has revealed an expanded role for these factors in safeguarding healthy cellular function in responding to stress outside of canonical DDR inducers. While checkpoint signaling is a critical component of the DDR, these signaling cascades must ultimately culminate in DNA repair, to avoid deleterious consequences. Phosphorylation of histone H2AX (referred to as γH2AX in the phosphorylated state) is a well-recognized and early marker for DNA damage and plays a critical role in connecting chromatin to DNA repair, by serving as a binding site for cellular repair machinery such as 53BP1. Furthermore, a number of DDR factors central to checkpoint induction are also responsible for the phosphorylation of γH2AX, including ATM and ATR [15]. An illustrative diagram of ATM, ATR, and DNA-PK signal transduction pathways is provided in Figure 1.

## 4. DNA Damage and DDR Signaling in PAH

The neoplastic theory of pulmonary arterial hypertension stems largely from observations of overlapping pathophysiological features of vascular cells from patients with iPAH and cancerous cells, and studies have demonstrated a role for the monoclonal expansion of endothelial cells (ECs) in the development of iPAH [16,17]. Accordingly, PAH ECs also demonstrate genomic instability and a predisposition to accumulate somatic mutations, and it has been demonstrated that PAH ECs derived from patients demonstrate increased mutagen sensitivity, suggesting impairments of endogenous DNA repair mechanisms [18]. In recent years, evidence for the involvement of DNA damage signaling in the role of genomic instability and aberrant cell survival has begun to accumulate. Markers of DNA damage from distal pulmonary arteries of PAH patients are increased compared to controls, and poly ADP ribose polymerase 1 (PARP1), an early DNA damage sensor and signal transducer, is upregulated, likely as a response to constitutive genomic insult. Indeed, PARP1 has demonstrated involvement in PAH pathogenesis, and PARP1 inhibition has therapeutic benefit in Sugen-hypoxia and monocrotaline rat models of PAH [19]. Furthermore, the ATM-CHK2 pathway has demonstrated involvement in a Sprague–Dawley lung recession/MCT rat model of PAH, in which ATM and its effector substrates demonstrated significantly increased activity during early disease development, but markedly reduced activity compared to controls in the advanced stages of PAH, suggesting the involvement of excessive DNA damage in the early stages of disease progression [20]. It has also been demonstrated that the inhibition of Chk1 elicits therapeutic benefit as Chk1 exerts a pro-proliferative state in PASMCs. Furthermore, elevated levels of Chk1 were seen in PASMCs isolated from the lungs of SIV infected macaques. It is suggested that chronic genomic insult due to long term oxidative stress and inflammatory states induced by infected may lead to the constitutive activation of Chk1 and potentiate a pro-proliferative PASMC phenotype that may contribute to the formation of PAH associated arterial lesions [21]. However, the role of Chk1 in endothelial cells during PAH pathogenesis may differ from that of PASMCs. Chk1 is known to act in concert with HSP90 to regulate endothelial nitric-oxide synthase (eNOS) [22], a critical factor in the regulation of vasoconstriction and relaxation. However, mitochondrial accumulation of HSP90 has also been demonstrated to support the cancer-like pro-survival and pro-proliferative states of PAH-PASMCs, an event which may be initiated by chronic oxidative insult, as is seen in the lungs of HIV-infected patients [23]. Furthermore, mitochondrial dysfunction and resulting increased DNA damage is also implicated in the pathogenesis of PAH through impaired oxidative stress response in PAH-PASMCs [24].

Genetic bone morphogenic protein receptor 2 (BMPR2) deficiency is associated with familial PAH, and the biology of BMPR2 signaling has provided insight as to molecular mechanisms driving endothelial dysfunction in the development of PAH [25,26]. It has been demonstrated that EC derived from BMPR2 deficient patients presenting with PAH are more vulnerable to DNA damage, and that this is a BMPR2 mediated mechanism that is intriguingly specific to endothelial cells, though this may be explained by the differential regulation of cell survival signaling by BMPR2 in ECs and SMCs. Moreover, it has been suggested that EC homeostasis is balanced along a BMPR2/BRCA1 axis, and that imbalance of this axis can potentiate the outgrowth of ECs with dysfunctional DDR mechanisms and aberrant pro-survival phenotypes. Indeed, BRCA1 expression has been observed to be attenuated in the endothelium of pulmonary arteries in patients with iPAH [27,28]. While BMPR2 mutations play an established role in hereditary PAH, the penetrance is relatively low (around 20%), suggesting that other genetic factors are involved in disease development [29]. Recent research utilizing whole exome sequencing and patient derived samples has identified genetic deficiency of another DNA damage response and repair factor as a risk factor for iPAH [30]. Topoisomerase binding protein 1 (TopBP1) is critical to checkpoint regulation and DNA damage repair by activation of ATR and rescue of stalled replication forks [31]. Mutations to the TopBP1 transactivation domain were identified in iPAH patients harboring no BMPR2 mutations. Following identification of this candidate gene, it was further demonstrated that TopBP1 levels are reduced in pulmonary microvascular endothelial cells derived from iPAH patients, and that TopBP1 deficiency is associated with impaired DNA repair and hypersensitivity to mutagens [30].

## 5. DNA Damage and DDR Signaling during HIV Infection

HIV is a small (9.2 kb) single stranded positive sense RNA virus that possesses an amazingly diverse repertoire of tools for the modulation and conditioning of its host’s micro and macro environments. While CD4+ T-cell cytotoxicity and depletion are the hallmarks of acute HIV infection, a large subset of cells (consisting primarily of resting T-cells and tissue resident macrophages) remain persistently infected [32,33,34]. HIV elicits pro survival and anti-apoptotic phenotypes in these cells, in order to facilitate long term infection and viral persistence, and HIV accomplishes this through numerous mechanisms, including the modulation of host cellular DDR factors which are central to cell cycle regulation and cell survival signaling [9,10,35]. HIV patients receiving HAART still display markers of systemic and localized inflammation, oxidative stress, and genomic instability although CD4+ T cell counts are normal and peripheral viral loads are low and even sometimes undetectable [36,37]. However, HIV persists in various reservoirs, and it is hypothesized that these reservoirs serve as sources of extracellular soluble viral factors, which are secreted by infected cells and known to elicit deleterious effects on uninfected bystander cells. Two proteins in particular, HIV Nef and Tat, have been the primary focus of research attempting to elucidate the mechanisms driving HIV-PAH. Both Tat and Nef are known to elicit pro-inflammatory and pro-oxidant states [38,39] and interact with host DDR factors to bolster viral replication and establish long term infection.

### 5.1. HIV Tat and the DDR

**Figure 2:**
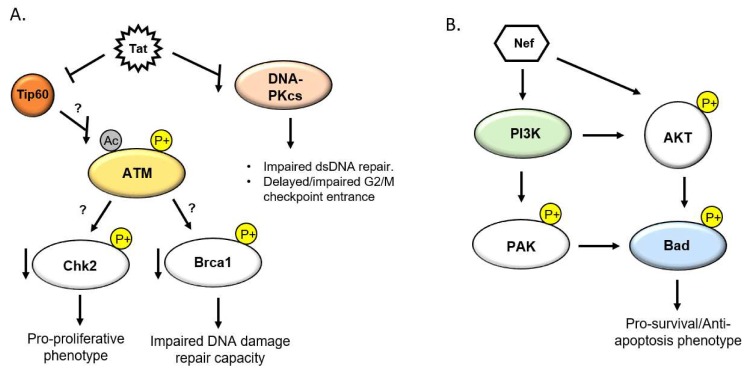
HIV factors Tat and Nef modulate the DNA Damage Response. (**A**) Tat is known to impair Tip60 activity, which in turn may attenuate ATM activation, resulting in impaired signaling to downstream effector substrates. Tat results in depression od DNA-PKcs and results in impaired dsDNA repair capacity and impaired checkpoint entrance ability. (**B**) Nef induces a pro-survival phenotype through PI3K and AKT activation, resulting in excessive phosphorylation of Bad and depression of apoptosis signaling.

The HIV transactivator of transcription (Tat) is a viral factor which is integral to the HIV life cycle by way of transactivation of pro-viral genes and the elongation of nascent viral transcripts [40]. A long-recognized property of Tat, however, is that it is actively secreted from infected cells into the extracellular milieu, where it is internalized by uninfected bystander cells [41,42]. Tat accomplishes this by means of a cell penetrating peptide in its basic core domain, which also contains a nuclear localization signal [43]. Once internalized, Tat traffics to the nucleus, where it is known to modulate a number of transcriptional and post transcriptional cellular processes [44,45]. Interestingly, Tat is detectable in the ng/mL range in sera of HIV-infected patients, despite treatment with HAART [42], and the amount of Tat detected does not appear to correlate with plasma viral load or CD4+ T cell count [46]. As such, Tat has been implicated in the pathological processes of a number of chronic HIV co-morbidities, including HIV-PAH [47]. In addition to its transcriptional activities, Tat interacts with and manipulates a number of host factors involved in DDR signaling. The histone acetyl transferase Tip60 (Tat interacting protein 60 kDa) was discovered as an interacting partner of Tat, though the cellular function of Tip60, as well as the biological importance of the Tat/Tip60 interaction, were unknown for years after its discovery [48]. Tip60 has since been demonstrated to be integral to DNA damage repair by regulating chromatin remodeling, gene transcription activation, and the activation of DDR factors [49]. It was later discovered that Tat inhibits the activity of Tip60 both through inhibition of the HAT domain as well as by facilitating its proteasomal degradation [10,50]. This ultimately results in a pro-survival cellular phenotype in which DNA damage fails to induce caspase mediated apoptosis. Moreover, it has recently been discovered that Tip60 is responsible for the activation of the aforementioned DDR master regulator ATM through the acetylation of lysine 3016. This event immediately precedes and is necessary for the auto-phosphorylation and dissociation/activation of ATM dimer subunits [51,52]. Tip60 is also involved in chromatin remodeling through the direct acetylation of histone H4, which results in chromatin relaxation and allowing access to DDR repair factors [15]. As such, Tat is poised to repress ATM-mediated DNA damage signaling through the Tip60/ATM axis, though more research is needed to explore this hypothesis (Figure 2A). Tat is additionally known to down regulate the DDR factor DNA protein kinase catalytic subunit (DNA-PKcs) [53], which is responsible for facilitating the repair of dsDNA breaks through non-homologous end joining (NHEJ). In turn, Tat results in increased DNA damage and impaired G2/M checkpoint transition ability (Figure 2A).

While Tat may predispose cells to the accumulation of DNA lesions and mutations by disrupting normal DDR function, it may also result in excessive DNA damage, by facilitating the production and accumulation of reactive oxygen species (ROS). It has long been recognized that HIV-infected individuals display increased biomarkers of chronic oxidative stress, including markers for oxidatively damaged genetic material such as oxidized nucleotides. Tat has been demonstrated to increase oxidative burden in the lungs of Tat transgenic mice, and it was recently discovered that Tat down regulates the critical oxidative stress response transcription factor nuclear factor (erythroid-derived 2)-like 2 (Nrf2) in human primary pulmonary arterial endothelial cells [38,54]. The. accumulation of excess ROS is one of the primary mechanisms of induction of dsDNA breakage and combined with potentially dysregulated DDR machinery, could position cells to accumulate deleterious somatic mutations over time.

### 5.2. HIV Nef and the DDR

HIV negative factor (Nef) is a HIV accessory protein that lacks enzymatic activity and is critical for immune evasion and establishment of viral persistence. However, Nef is also heavily implicated in the pathogenesis of HIV-PAH and other HIV associated cardiovascular diseases [55]. Nef has been shown to be transferred into coronary endothelial cells from HIV-infected or Nef transgenic T-cells, and has additionally been detected in the pulmonary endothelium of HIV transgenic mice and SIV/HIV Nef chimeric infected macaques [56]. In a macaque model of SIV/HIV associated PAH, Nef was detected in the endothelial layer of complex vascular lesions associated with the development of PAH [57], suggesting a role for Nef in the pathogenesis of HIV-PAH. While Nef lacks the cellular transduction properties of Tat, it is known to be secreted in extracellular vesicles [58] and is often detectable in the plasma of patients with receiving anti-retroviral therapy [59]. Furthermore, Nef is detected in the peripheral blood mononuclear cells of HIV-infected patients, the majority of which are uninfected [60]. The extravasation of infected T-cells and macrophages across the endothelium and into the periadventitial space represent another potential contributing source of Nef. Nef supports viral replication and persistence, not only by facilitating immune evasion, but also by inducing an anti-apoptotic and pro-proliferative cellular state. Phosphoinositide-3-kinase (PI3K) and AKT serine/threonine kinase (AKT) signaling is intricately tied to the DNA damage response and maintenance of cellular homeostasis, in response to genotoxic stress [61]. In particular, PI3K/AKT signaling is a prominent and well-studied pathway involved in the regulation of apoptosis. Under physiological conditions, PI3K mediated phosphorylation of AKT results in an AKT signaling cascade which induces an anti-apoptotic state through the phosphorylation and inhibition of pro-apoptosis Bcl-2 family members (Figure 2B) [9]. In response to DNA damage, AKT interacts interchangeably with members of ATM/ATR mediated DDR factors, including BRCA1, CHK1, and CHK2, and facilitates dsDNA damage repair via the activation of NHEJ and repression of homologous end joining homology directed repair [61,62]. In doing so, AKT elicits a pro-survival phenotype by the modulation of the aforementioned factors. PI3K/AKT and DDR signaling is a carefully balanced act, and over activation of AKT is associated with a plethora of cancer-like processes and cancerous malignancies. It has been demonstrated that extracellular Nef is internalized by T cells and activates AKT through a PI3K dependent mechanism, resulting in the induction of anti-apoptotic and pro-proliferative signals [9]. Additionally, it has been demonstrated that Nef also interacts with PI3K to elicit a pro-survival phenotype, but in an AKT independent manner (Figure 2B) [63]. Through the modulation of PI3K/AKT signaling, Nef likely serves to maintain cell survival in the face of excessive DNA damage generated by the HIV lifecycle and other viral factors, which directly modulate DDR machinery and also result in the accumulation of excess DNA damaging free radicals. Taken together, it is reasonable to postulate that Tat and Nef act individually and in concert in pulmonary vascular bystander cells to induce a pro-survival and ant-apoptotic phenotype, conducive to the accumulation of DNA damage induced mutations genomic instability observed in PAH.

## 6. Discussion

The prevalence of PAH in the HIV-infected population is 2000 times greater than in the general population [47]. Although the efficacy of HAART in reducing the risk of developing HIV-PAH remains controversial, the overall prevalence of HIV-PAH appears to be largely independent of the global implementation of HAART, with little evidence to suggest that the implementation of anti-retroviral therapy alone is sufficient to treat HIV-PAH. Although drug–drug interactions must be taken into consideration during the treatment of HIV-PAH, the overall management of the disease is similar to iPAH. The WHO recommends treatment of HIV infection with combination anti-retroviral therapy regardless of CD4+ T- cell count, however, therapeutic benefit of HAART alone on HIV-PAH is a contentious topic requiring further research. Current HIV-PAH and iPAH therapies, including endothelin receptor antagonists and phosphodiesterase inhibitors, are largely mediators of vasodilation/vasoconstriction, as well as smooth muscle and endothelial cell proliferation [64], although many more clinical studies into the efficacy of these therapies in HIV-PAH patients compared to iPAH patients are greatly needed. While the cellular physiological processes such as hyper-proliferation and apoptosis resistance are similar, the molecular pathways orchestrating these phenotypes may be disparate in HIV-PAH patients, compared to those in HIV naïve PAH affected individuals. Interestingly, while defects in appropriate DDR signaling are heavily involved in a plethora of diseases, the deficiency in DNA repair capacity and ability to maintain short- and long-term genomic stability are exploitable features for therapeutic intervention. Cells which have gained a proliferative selective advantage through the loss of any branch of the DDR exist on the precipice of total genomic collapse, due to a paucity of redundant DNA maintenance mechanisms. Indeed, many chemotherapies for malignant diseases in which DDR signaling is heavily dysregulated take advantage of these diseased cellular properties, by inhibiting certain branches of the DDR and tipping the scale towards an unsustainable cellular genetic landscape [65]. As such, the potential exists to exploit similar mechanisms in pulmonary vascular endothelial cells, that demonstrate similar genetically unstable hyper-proliferative phenotypes through somatic mutation or exposure to soluble HIV factors. Interestingly, histone deacetylase inhibitors (HDACi) have demonstrated therapeutic benefits in the animal models of PAH [66], and HDACs play a pivotal role in DNA damage signal transduction. Inhibition of HDAC6, known to be upregulated in the lungs and distal pulmonary arteries of PAH patients, was seen to reduce PASMC proliferation as well as sensitize PAH vascular cells to apoptosis through activation of the Ku70 subunit of the DNA-PK complex [67]. Furthermore, anti-neoplastic agents typically used in the treatment of cancer have been shown to mitigate pulmonary vessel thickening through the targeted killing of vascular cells in a hypoxic rat model of PAH [68]. In particular, the DNA damaging anthracycline compound daunorubicin displayed remarkably specific cytotoxic activity against vascular cells in remodeled vessels, while sparing the same cell types in control animals, suggesting an inherent sensitivity of pathological cell types to the DDR mediated apoptotic effects of daunorubicin [69].

## 7. Conclusions

More research is warranted to elucidate the role of HIV modulation of DDR signaling in the pathogenesis of HIV-PAH, as well as the role of DDR factors in iPAH. Transgenic animals provide an opportunity to observe the effects of individual viral factors or multiple candidate factors in concert, while primate models infected with HIV/SIV chimeric viruses will allow researchers to observe the physiological consequence of these putative perturbations to the DDR in the pulmonary vasculature. Overall, the intersections of PAH, DNA damage response signaling, and chronic HIV infection provide unique opportunities to elucidate novel mechanisms of PAH pathogenesis, with implications beyond HIV-PAH alone.

## Abbreviations

DDR	DNA damage response
EC	Endothelial cell
HAART	Highly active anti-retroviral therapy
HIV	Human immunodeficiency virus
HIV-PAH	HIV-associated pulmonary arterial hypertension
iPAH	Idiopathic pulmonary arterial hypertension
MPAP	Mean pulmonary arterial pressure
NHEJ	Non-homologous end joining
PAEC	Pulmonary artery endothelial cell
PAH	Pulmonary arterial hypertension
PASMC	Pulmonary artery smooth muscle cell
PAWP	Pulmonary artery wedge pressure
PVR	Pulmonary vascular resistance
ROS	Reactive oxygen species
SIV	Simian immunodeficiency virus
SMC	Smooth muscle cell
